# Healthcare waste generation and management practice in government health centers of Addis Ababa, Ethiopia

**DOI:** 10.1186/1471-2458-14-1221

**Published:** 2014-11-25

**Authors:** Menelik Legesse Tadesse, Abera Kumie

**Affiliations:** Menelik II Health Science College, Addis Ababa, Ethiopia; Department of preventive medicine, School of Public Health, Addis Ababa University, Addis Ababa, Ethiopia

**Keywords:** Healthcare wastes, Health centers, General waste, Hazardous waste, Addis Ababa, Ethiopia

## Abstract

**Background:**

Healthcare wastes are hazardous organic and inorganic wastes. The waste disposal management in Addis Ababa city is seen unscientific manner. The waste management practice in the health facilities are poor and need improvement. This study will help different organizations, stakeholders and policy makers to correct and improve the existing situation of healthcare waste legislation and enforcement and training of staff in the healthcare facilities in Addis Ababa. The study aimed to assess the existing generation and management practice of healthcare waste in selected government health centers of Addis Ababa.

**Methods:**

The cross-sectional study was conducted to quantify waste generation rate and evaluate its management system. The study area was Addis Ababa. The sample size was determined by simple random sampling technique, the sampling procedure involved 10 sub-cities of Addis Ababa. Data were collected using both waste collecting and measuring equipment and check list. The Data was entered by EPI INFO version 6.04d and analyzed by and SPSS for WINDOW version15.

**Results:**

The mean (±SD) healthcare waste generation rate was 9.61 ± 3.28 kg/day of which (38%) 3.64 ± 1.45 kg/day was general or non-hazardous waste and (62%) 5.97 ± 2.31 kg/day was hazardous. The mean healthcare waste generation rate between health centers was a significant different with Kurskal-Wallis test (*χ*^*2*^ = 21.83, p-value = 0.009). All health centers used safety boxes for collection of sharp wastes and all health centers used plastic buckets without lid for collection and transportation of healthcare waste. Pre treatment of infectious wastes was not practiced by any of the health centers. All health centers used incinerators and had placenta pit for disposal of pathological waste however only seven out of ten pits had proper covering material.

**Conclusion:**

Segregation of wastes at point of generation with appropriate collection materials and pre- treatment of infectious waste before disposal should be practiced. Training should be given to healthcare workers and waste handlers. Incinerators must be constructed in a manner that facilitates complete combustion and the lining of placenta pit should be constructed in water tight material.

## Background

In developing countries such as Ethiopia the international or local policy that generator of waste is responsible for the proper management, treatment and disposal of waste has remained on paper and is yet to be implemented. The notion that waste is the responsibility of the government authorities has not enable waste generators to appreciate the negative impact of improper waste disposal. Waste is generated from any where such as home, school, industry and health care facilities.

Healthcare wastes (HCW) that are generated from healthcare establishments; hospitals, health centers, medical research centers, pharmaceutical manufacturing plants, pharmacies, blood banks, and home health care activities are some of the generators of healthcare waste. Healthcare wastes generated from health facilities and diagnostic activities can be broadly categorized to general waste and hazardous waste. However, it remains true only when proper segregation and separation of waste is practiced [1].

World Health Organization (WHO) reported that from the total waste generated by health care activities, 85% is general waste and the balance is considered as hazardous, as it tends to be infectious, toxic or radioactive [2].

Study in Ethiopia showed the mean standard deviation (± SD) of healthcare waste generation rate per health center was 1.79 ± 0.57 Kilogram/day (kg/day). Of which (52.0%) 0.93 ± 0.3 kg/day and (48.0%) 0.86 ± 0.33 kg/day were general or non-hazardous waste and hazardous waste, respectively [3]. Another study in the hospital of Addis Ababa, Ethiopia showed the mean healthcare waste produced were 0.5 kg/patient/day and 1.6 kg/bed/day [4]. There are different estimates regarding the share of general and hazardous constituents of health care waste generation. This may be the segregation of healthcare waste at point of generation is weak in health facilities and assumed these wastes are non-contaminated and pose no risk of infection. Such management of the healthcare waste is doing by traditional way and some of the healthcare waste disposal to be in-forced by the good will of managers. Another assumption is the limitation of existing facilities, lack of adequate institutional arrangements, operation insufficiency, local authorities inefficiency in performing their task effectively are some points for the poor management but few take proper care of their waste.

Now wastes threaten the public, since the health care facilities are situated in the heart of the city and therefore healthcare waste, if not properly managed can cause dangerous infection and posses a potential threat to the surrounding environment, person handling it and to the public.

Health and environmental effects, uncertainty regarding regulations and negative perceptions by waste handlers are some important concerns in healthcare waste management in a country [5].

There were limited studies in our country as focused on the hospital healthcare waste generation and handling practice. At present, there is no available information that describes the actual practice of handling the healthcare waste in the health centers of Addis Ababa. Effective management of proper disposal of the healthcare waste is uncertain. This study focus on the health center (HC) because they are the primary health care unites in the city. Addis Ababa City Administration Health Bureau (AACAHB) in the near future builds health centers in every Woreda also the health policy of the country supports the healthcare waste management. So the outcome of the study can be used as base line data for planning and implementation activities. It can also help the policy makers, the researchers and other concerned bodies to develop effective healthcare waste management system to Addis Ababa and the country as a whole.

### Objectives

#### General objective

To assess the existing generation and management practice of healthcare wastes in selected government health centers of Addis Ababa.

#### Specific objectives

To determine the healthcare waste generation rate at selected government health centers.

To describe the type of healthcare waste generated from selected government health centers.

To assess the practice of waste management at selected government health centers.

## Methods

### Study design, area and population

**Figure 1:**
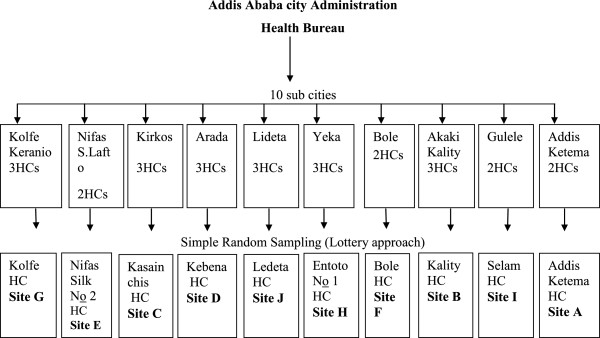
**Schematic representation of each Health Centers in Addis Ababa city Administration, January 2011.**

Institutional based cross-sectional study was conducted to quantify healthcare waste generation rate and evaluate its management system from 25^th^ to 31^st^ of January 2011 in the governmental health centers of Addis Ababa. Addis Ababa is the capital city of Ethiopia, with the total population of 2,917,295 (1,389,817 males and 1,527,478 females) [6]. The city has three layers of administration; the City Administration at the top, 10 Sub cities Administration in the middle and 116 Woredas at the bottom [7]. Each sub city administration has the estimated population of 300,000 and each Woreda administration has the population of 30,000. The increase in population number favors the health facilities to have more healthcare wastes. The source population was 26 government health centers in 10 sub cities [8]. The study population, 10 health centers, 1 from each sub city was selected by simple random sampling and lottery approach was employed (Figure 1).

### Sample size and sampling procedure

An estimating a mean sample size for a very large population formula was used to calculate the sample size [9]; considering an assumption of proportion 95% level of confidence and 7% marginal error. This was done by referring from previous studies mean generation: 1.79 kg per day; SD 0.57 kg per day over 80 measurements in 10 hospitals [3]. Seven consecutive days sample measurement was done in ten health centers. Data were collected by using pre-prepared data entry sheet for the daily measurement of the amount of healthcare waste rate, pre tested check list and interview tools. Data were collected for 24 hours from each sampling unit in different color coded polyethylene bags. The bags color was yellow, red and black. Yellow bags were contained for infectious clinical wastes and yellow safety boxes for sharp wastes. Black bags were contained for non infectious (non risk) wastes. Red bags contained for drugs and radioactive hazardous wastes. Each plastic bag were labeled by BANNER brand sticker showed the date, category of waste, room for waste collected and time started. Total wastes per day were measured at each study unit by removing the plastic bags every morning and its weight was measured every day at 9:00 A.M using weighing scale.

The site visit was conducted by using check list to review of segregation, handling, collection and storage practice at the various case teams of the health centers. Interview was conducted about the management issues with the health center managers. Care was taken during waste collection; data collectors use gloves, masks, gown and antiseptics to prevent infection. At least 11 case teams were chosen; Delivery, Emergency Injection and Dressing room, Laboratory, Human immunodeficiency Virus (HIV) Counseling and Testing and Anti Retroviral Treatment (ART), Pharmacy, Focus Antenatal Care (FANC), Expanded Program for Immunization (EPI), Family Planning (FP), Tuberculosis (TB) and Leprosy, Out Patient Department (OPD), and Integrated Management of Neonatal and Child Illness (IMNCI) and growth monitoring. Incinerator and deep burial and placenta pits were seen.

Measurement was done by using Weighing Scale which is analog and digital is used at the study units and recorded. Data quality was assured by the measurement of waste by using spring balance capacity range from 20gm to 400 gm, Electronic infant Scale model ACS-20A-YE, Electronic balance model Sartorius Basic Type BA6100, Electronic compact balance model EPB-10001 L digital scale and XY Electronic balance model XY-JC/JB”. The measuring instruments were calibrated by using known weight, a standard of 20 g, 100 g, 500 g and 1000 g weighting objects every morning before the actual measurement started. The standard value was recorded for comparison to the daily activities. For the purpose of data collection 10 enumerators and 3 supervisors were take one day training by the principal investigator.

### Variables

The amount of healthcare waste generated and waste management practice were the outcome variables where as the material used for healthcare waste collection, transportation, presence or absence of healthcare waste management policies and segregation of healthcare waste at the source were independent variables.

### Statistical methods

Data were entered to Statistical Software for Epidemiology (EPI INFO) version 6.04d and analyzed by Statistical Package for Social Science (SPSS) for Window Version 15 to enable the estimation of healthcare waste generation rate in each health center. Comparison of visitors, healthcare waste generation rate and its types among health centers were compared using Kurskal-Wallis test and relation of visitors and amount of healthcare waste in study health centers were computed by Spearman’s rank correlation. The results on evaluation of the average quantity of healthcare wastes and waste management system were reported using different descriptive statistics.

### Ethical consideration

Ethical clearance was obtained from Addis Continental Institute of Public Health (ACIPH); Permission was obtained from Addis Ababa city Administration Health Bureau and from the Managers/Directors of the health centers. Consent was obtained from each health centers. The participants were free either to participate or not. The study wasn’t cause any harm to neither the study subjects nor to the data collector. Data collectors were trained to use protective materials when handling healthcare wastes. Supervisors were alert if there were any injuries during collection period.

## Results

### Service, case team and patient flow in the study health centers

A total of 22,045 patients visited in all case teams, of which 4,647(21.08%) patients visited OPDs in all health centers in 7 days. The mean (±SD) patient flow per day in all sections and the mean patient flow at OPD were 316.6 ± 104 and 66.9 ± 23.97, respectively. More patients were seen to Site E and Site G health centers, 3864 and 3041, respectively. On the other hand less number of patients was seen to Site J and Site H health centers, 1396 and 1641, respectively.

### Waste Generation rate

#### Daily HCW generation in health centers

The mean (±SD) healthcare waste generation rate was 9.61 ± 3.28 kg/day, of which 3.64 ± 1.45 kg/day (38%) was general waste and 5.97 ± 2.31 kg/day (62%) was hazardous waste. High amount of healthcare waste per day was generated at Site G and Site E health centers, 14.49 kg/day and12.55 kg/day, respectively. Small amount of healthcare waste was recorded at Site J and Site D health centers, 3.95 kg/day and 5.5 kg/day, respectively (see Table 1).

**Table 1:** **The amount of daily Healthcare waste generation rate in health centers, Addis Ababa City Admin., January 2011**

Name of health center	Healthcare waste, Kg/day			
	Total HCW in 7 days	Mean of HCW mean (+ SD)	Mean of general waste (%)	Mean of hazardous waste (%)
Site A	77.41	11.06 ± 4.49	5.50(49.76)	5.56(50.24)
Site B	70.92	10.13 ± 5.17	2.33(22.98)	7.80(77.02)
Site C	44.55	6.36 ± 3.83	2.93(46.02)	3.44(53.98)
Site D	39.18	5.597 ± 3.75	1.81(32.39)	3.78(67.61)
Site E	87.83	12.55 ± 6.63	3.79(30.23)	8.75(69.77)
Site F	74.81	10.69 ± 5.52	3.196(29.9)	7.49(70.1)
Site G	101.40	14.49 ± 5.28	5.81(40.09)	8.68(59.91)
Site H	76.40	10.91 ± 5.49	4.35(39.86)	6.56(60.14)
Site I	72.50	10.36 ± 5.40	4.78(46.15)	5.58(53.85)
Site J	27.62	3.95 ± 2.48	1.89(47.99)	2.052(52.01)
Overall mean	67.26	9.61	3.64	5.97
SD	22.97	3.28	1.45	2.31

The types of hazardous waste generated from study health centers were sharps, infectious and pathological which were placenta and blood. The mean (±SD) generation rate of sharps, infectious and pathological waste in each health center was 0.87 ± 0.28 (14.57%), 2.2 ± 0.84 (38.36%) and 2.8 ± 1.4 (47.24%) kg/day, respectively (see Table 2).

**Table 2:** **Distribution of types and amount of daily hazardous and non-hazardous waste generation rate in health centers, Addis Ababa City Admin., January 2011**

Name of health center	Sharps Kg/day	Infectious Kg/day	Pathological Kg/day	Total hazardous waste Kg/day
Site A	0.441	2.043	3.072	5.556
Site B	1.086	2.462	4.256	7.804
Site C	0.584	1.343	1.508	3.435
Site D	0.745	1.385	1.654	3.784
Site E	1.148	3.587	4.019	8.754
Site F	1.120	3.088	3.283	7.491
Site G	1.213	2.423	5.043	8.679
Site H	0.940	2.817	2.807	6.565
Site I	0.861	2.756	1.961	5.577
Site J	0.530	0.971	0.551	2.052
Average	0.867	2.287	2.815	5.969
SD	0.2802	0.8419	1.4018	2.3095

### Daily HCW generation rate in different case teams

In different case teams the amount of healthcare waste generation rate was different. The mean (±SD) healthcare waste generation rate in all section was 8.66 ± 10.95 kg/day. Great amount about (40.79%) 38.60 ± 2.0 kg/day of healthcare waste was from delivery case team where as less amount (0.97%) 0.915 ± 0.97 kg/day of healthcare waste was generated at IMNCI case team. The mean health care waste generation rate in different case teams in the study health centers was statistically significant (*χ*^*2*^ = 19.62, p-value < 0.033) (see Table 3).

**Table 3:** **Distribution and daily amount of healthcare waste generation rate by point source in Health centers, Addis Ababa City Admin., January 2011**

Case teams	HCW (Kg/day) Mean ± (SD)	Percent	Mean Rank
OPD	3.029 ± 0.15	3.20	8.67
Pharmacy	12.40 ± 0.65	13.10	31.00
Laboratory	11.586 ± 0.5	13.11	24.25
Emergency	12.406 ± 0.5	13.11	25.33
FNAC	2.759 ± 0.16	1.37	11.67
Delivery	38.600 ± 2.03	40.79	25.00
TB	1.973 ± 0.14	2.09	8.67
EPI	3.055 ± 0.16	3.23	11.67
FP	4.950 ± 0.2	5.23	16.33
VCT & ART	3.597 ± 0.25	3.80	13.67
IMNCI	0.915 ± .097	0.97	6.00
Mean	8.66		
SD	10.95		

*X* ^2^ = 19.62, p-value < 0.033 degree of freedom = 10.

### Annual HCW generation rate estimation

The annual healthcare waste generation rate can be calculated and the estimation per health center was 3501.86 ± 1204.29 kg/year. The annual flow of patients and mean healthcare waste generation rate per patient per day (the assumption was each patient who visited the health center may generate the same amount of HCW throughout the year).

### Visitors and HCW generation comparison

Patient flow, healthcare waste generation rate and its types such as general and hazardous waste (sharps, infectious, and pathological waste) among different health centers were compared using Kruskal-Wallis test to check for the presence of significant difference among their values. There was a significant difference to mean of healthcare waste (*χ*^*2*^ = 21.83, p-value = 0.009) and the mean hazardous waste (*χ*^*2*^ = 26.75, p-value = 0.002) among study health centers. There was no significant difference for the mean patient flow (*χ*^*2*^ = 14.504, p-value = 0.106) and the mean general waste (*χ*^*2*^ = 13.41, p-value = 0.145) (see Table 4).

**Table 4:** **Comparison of visitors, HCW generation rate and its types among health centers, Addis Ababa City Admin., January 2011**

Name of health center		Mean rank		
	Patient flow	Total HCW	General waste	Hazardous waste
Site A	36.79	42.57	46	38
Site B	31.21	38.14	27	42.71
Site C	36.57	25	31.57	20.29
Site D	33.43	20.14	21.71	22.14
Site E	53.71	44.43	38.86	48.86
Site F	38.36	41	33.86	44
Site G	46.86	51.86	47.14	51.86
Site H	27.43	40.43	42.14	42.43
Site I	32.07	37.86	43.43	32.43
Site J	18.57	13.57	23.29	12.29
Chi-Square	14.504	21.825	13.414	26.751
Asymp. Sig.	0.106	0.009	0.145	0.002

Degree of freedom = 9.

The extent or strength of linear relationship between numbers of patients and amount of healthcare waste generation rate were checked using Spearman’s rank correlation coefficient (rs) in all health centers. Spearman’s rank correlation coefficient showed that there was a positive linear relationship as number of patients increased healthcare wastes also increased in all study health centers. A strong linear relationship was observed at Site D, Site C and Site J health centers, the spearman’s correlation coefficient was 0.964, 0.955 and 0.929, respectively. Site B, Site E and Site H health centers were far from a perfect linear relationship 0.071, 0.697, and 0.500 respectively (see Table 5).

**Table 5:** **Relation of visitors and amount of healthcare waste in study health centers, Addis Ababa City Admin., January 2011**

Name of health center	Spearman’s rank correlation coefficient
Site A	0.857
Site B	0.071
Site C	0.955
Site D	0.964
Site E	0.697
Site F	0.875
Site G	0.847
Site H	0.500
Site I	0.857
Site J	0.929
Total	0.685

### Waste management

All health centers used different color plastic buckets for the collection healthcare waste. Half of the health centers didn’t have separate containers for the collection of hazardous and non hazardous wastes. These practices were adjacent to the study area that half of the study health centers had no formal or informal separation of the healthcare waste guidelines (Table 6). Moreover the labeling of the waste containers didn’t see by seven of the study health centers. Three out of ten health centers, the waste handlers didn’t wear heavy duty gloves and wear sturdy shoes while working. All health centers used open bucket for the transportation of healthcare wastes (Table 6).

**Table 6:** **Healthcare waste management practice and risks of healthcare waste in study health centers Addis Ababa city Administration, January 2011**

Description	Yes	No
	n = 10	n = 10
Separate containers for hazardous and non hazardous waste	5	5
Formal or informal HCW separation guideline	5	5
Labeling of the container	3	7
Personal protective equipment usage by HCW handlers	7	3
HCW transportation container with lid	0	10
Presence of interim HCW storage container	9	1
Treatment of infectious waste before disposing off	0	9
Ash remain disposal within close damping	8	2
Fencing the incinerator	7	3
Placental pit constructed with concrete	7	3
Focal person for HCW in Health center	10	0
Presence of SOP FOR HCW	6	4
Presence of HCW management committee	3	7
Registration book for any HCW injury or contamination	6	4
Managers concern on HCW	6	4
Needle stick injury in the past 12 months	8	2
Any risk to HCW handlers	7	3

Pathological wastes were seen to all studied health centers. Cytotoxic to three of the health centers and reagent wastes to four health centers were found. The out dated pharmaceuticals found by four of the health centers were waiting decision for disposal.

The majority, nine of the health centers, had interim waste storage and easy for the staffs to access but six of the health centers interim waste storage containers had no lid and the storage time was over 48 hours (Table 6).

Treatments of wastes by all health centers were unthinkable. All health centers used incinerators for onsite destruction of health care wastes except placenta which was disposed to placenta damping pits. The incinerators were built from local bricks but four health centers have no adequate air inlets for facilitating combustion of wastes. The ash remains for eight health centers had placed at the bottom of the incinerators to be stored and two of the health centers had kept to the open field. The incinerators were fenced by seven of health centers. Five of the health centers incinerator, ash disposal parts and placenta pits were away from any of the water source. Three out of ten health centers the placenta pits were not fulfilled the standard of WHO where their slabs made from stone and wood and the coverage of the pits were fenced by plastics with no cautioned signs to protect the workers and other clients (Table 6).

The responsibility of healthcare wastes showed that eight of the health centers had different health professionals and administrator staffs to run the management, two health centers had used sanitarians.

Six of the health centers had no current standard operational procedures for healthcare waste management, it was also confirmed by seven of health centers haven’t had any applicable national, regional and local guideline for health care wastes management moreover seven of the health centers didn’t organize healthcare waste management committee (Table 6).

### Healthcare workers related to HCWs risk

Six out of ten health centers management had no concern about the healthcare waste management as their routine work while seven health centers managers agreed that healthcare wastes pose any risk to their waste collectors, handlers and healthcare workers. In this study there were at least 48 waste handlers worked to ten health centers among these eight managers knew the waste handlers were encounter needle stick injury in the past 12 months (Table 6). All injuries were occurred in the work hours, the types of injuries sustained were deep injury, slight skin, superficial and splash answered by of the health center managers. Four of the health centers had no registration book for any injury or healthcare waste contamination to their staffs (Table 6).

## Discussion

The mean (±SD) healthcare waste generation rate per health centers was 9.61 ± 3.28 kg/day, of which (38%) 3.64 ± 1.45 kg/day was general or non-hazardous waste and (62%) 5.97 ± 2.31 kg/day was hazardous. There was a significant difference (*χ*^*2*^ = 21.83, p-value = 0.009) in the total healthcare waste generation rate between health centers (see Table 4). This may be due to higher visitors’ number flow in season of the year and resource allocation to the health centers. Mean while there was no significance different (*χ*^*2*^ = 14.504, p-value = 0.106) in patient flow between study health centers.

There was statistically significant of healthcare waste generation rate in different case teams of the health center (*χ*^*2*^ = 19.62, p-value <0.033). The highest generation rate 40.79% of healthcare waste was in delivery case team where as fewer amounts 0.97% of healthcare waste was in IMNCI and growth monitoring case team (Table 3). This variation may be due to the difference of number of attendance, the kinds of healthcare service, the type and the nature of waste generated at each case team.

The annual mean (±SD) healthcare waste generation rate per health center was 3501.86 ± 1204.29 kg/year. The assumption was the preferable method to estimate annual health care waste generation rate because the mean of annual healthcare waste was determined by annual patient flow within the health center.

The mean healthcare waste generation rate in gram per patient per day per health center in this study was 57.37. It was higher than the study done in Ethiopia 35 g/patient/day in health center and 500 g/patient/day in hospital [3]. It was also different from another study done in Sylhet city, Bangladesh in diagnosis center and higher clinics, the mean healthcare waste generation rate was 0.041 kg/patient/day [10]. This variation may be due to geographical location, season of the year, availability of different facilities, social status of the patients (i.e. income, living standard, awareness about disease), healthcare waste management and legislation of system of the country.

The proportion of general (38%) and hazardous (62%) of healthcare wastes in this study was different in WHO report in hospital setting, general was 85% and hazardous was 15% [2].

It was also different with the study done in Sylhet city, Bangladesh in diagnosis center and higher clinics general waste accounted 63.97% and hazardous waste accounted 36.03% [11]. The difference could be due to seasonal variation, availability of different facilities, resource allocation and the variation of denominators between hospitals and health centers.

All health centers used safety boxes for collection of sharp wastes. It was better than the study done in Ethiopia on injection safety, safety box was observed only in 2(4%) of the 52 health facilities assessed [12]. This variation may be due to the risk of used needles and sharps related with improper collection might be given better attention by governmental health system, getting training by waste handlers and managers.

Waste segregation and treatment are the most important option in the management of hazardous wastes. Waste management system in this study revealed that segregation of waste at source was practiced by half of health centers. This finding was most likely consistent with the survey conducted on four federal hospitals by Ministry of Health (MoH); all but one hospital was segregate infectious waste at source [13]. This was similar with other study conducted on four hospitals in Nigeria segregation of waste was not practice by any of the study health institutions [14].

In this study all the health centers used incinerators, no open burning was occurred for disposing used needles and other sharps, different studies had done in most African countries, waste disposal was reported to be problematic. Cameroon (1998), Chad (1997), Coted’Ivoire (1997), Guinea-Bissau (1997), and Uganda (1998) showed that no health centers had the facilities for safe disposal of used needles and other sharps. In Ethiopia (1997-98), Kenya, Rwanda and Zambia, incineration of used syringes and needles was reported to the common practice [15]. Another study conducted by Yoseph in similar setting revealed that 42.5%( 17 out of 40) of the health institutions incinerators were used for disposing used needles and other sharps and the rest 57.5% of the institution used open burning and other methods to dispose used needles and other sharps [16].

In this study all health centers used sewer lines for liquid waste from laboratory and delivery room. It was simply dispose without any treatment in the premises of the health centers. This was similar with the study done by MoH in Ethiopia in 1989 in 16 health centers and 48 clinics. It was reported that most of them had no proper liquid waste and solid waste disposal facilities [17].

**Figure 2:**
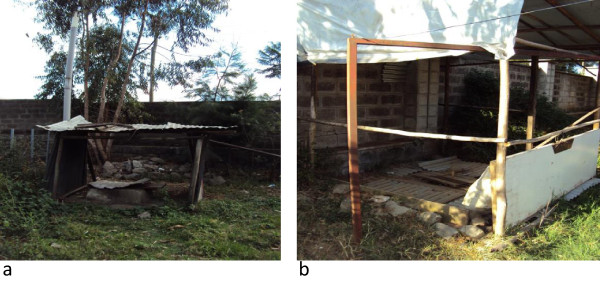
**Placenta pits (a. slab made from concrete; b. made from local wood).**

Three out of ten studied health centers the placenta pits had not been found constructed by concrete foundation (Figure 2a and b). They were not convenient to waste handlers, the public and the environment by releasing bad odor to the atmosphere and accessible for vector breeding.

Segregation of wastes at source was not practiced at all health centers in this study and also in West Gojjam zone, Ethiopia [3].

Eight out of ten health centers in the study area showed that there were needle stick injury, different studies showed in developing countries the data available are very few and are mere gross under estimation of the real risks [2]. This needle stick injury may be related with improper handling of healthcare wastes particularly sharps because unsafe sharps waste collection due to improper segregation of wastes at the source. Sharps and needle stick injuries are the commonest form of HIV, Hepatitis B Virus and Hepatitis Virus C exposure in health institutions especially for healthcare workers and waste handlers.

Ministry of Health of Ethiopia has prepared healthcare waste management guidelines in 2007 for the safe handling and disposal of health care wastes also the promotion of occupational health and the protection of the environment from healthcare waste [18]. But six out of ten studied health centers, Standard Operational Procedures, as well as any applicable local or regional guidelines about healthcare waste management were not found. Seven out of ten health centers didn’t organize safety committee to follow the disposal of HCWs. It was similar with a review done by Solomon, on healthcare waste management in Ethiopia, it was reported that there was no guidelines specifically deals with hazardous waste and waste from healthcare activities at micro level even though there is at federal level prepared by the Environmental policy of Ethiopia, the Public Health Proclamation No.200/2000 Federal Democratic Republic of Ethiopia and the Environmental Pollution Proclamation No.300/2002 [12]. The existing of gap among the study groups may be due to less attention is given on healthcare waste management by responsible authority such as sub cities health offices that are responsible for any wastes management, lack of supervision with the responsible body on Addis Ababa city Government Health Bureau and lack of healthcare waste management committee at Federal Ministry of Health.

## Conclusions

Healthcare waste management system had been given very little attention in all health centers. Segregation and treatment of healthcare waste were not well practiced this exposes healthcare workers, waste handlers and the public to health risk. Puncture and leak proof containers with a lid should be used for disposal in order to minimize the risks for healthcare wastes. Pretreatment of infectious waste and liquid waste must be practiced before disposing to the sewer and environment. Incinerators must be fenced and the lining of placenta pits must be constructed in water tight materials. The other important point should be training on healthcare waste management for waste handlers and healthcare workers bring greatest change on practice and management of healthcare waste. Healthcare waste management committee must be established at all level of the health facilities and preparation of National Guideline for Healthcare Waste is encouraging. The other option should be outsourcing healthcare wastes to the private partners or other stake holders also important. The last not least further research on healthcare waste generation rate and management at different seasons is strongly recommended.
